# Who are leeches? Exploring malleability in human–leech relations through ethnographies from Dagestan and Turkey

**DOI:** 10.1186/s13002-025-00801-4

**Published:** 2025-07-17

**Authors:** Iwona Kaliszewska, Iwa Kołodziejska

**Affiliations:** Faculty of Culture and Arts, Institute of Ethnology and Cultural Anthropology, University of Warsaw, Ul. Żurawia 4, 00-503 Warsaw, Poland

**Keywords:** Leech, Malleability, Reversibility, Communication, North Caucasus

## Abstract

This paper introduces the concept of "malleability" as a lens for understanding human interactions with writhing animals, using leech–human relations as a case study. Our research is based on ethnographic fieldwork among Dagestani healers in Dagestan and Turkey, 2019–2024. We argue that the malleability of leeches influences leeches’ capacity for reversibility and shapes human–leech communication through their physical transformations and social roles. Through their flexible bodies, leeches enable nuanced, multisensory exchanges, influencing how humans interpret their actions—whether as cooperative, resistant, or purposeful behaviour. Malleability also mediates human sensory and emotional responses, evoking reactions ranging from disgust to admiration. Our findings reveal that leeches occupy a spectrum of roles in human perception and practice, serving as near-companions or ethical subjects, despite the lack of bioethical protections for their use in research, and as commodities or tools in medical contexts. Their physical and semantic malleability enables this fluid reversibility in human perceptions and practices. Methodologically, we advocate for “immersive duo-ethnography”, which incorporates the researchers' bodily experiences as tools for examining non-verbal interactions between writhing animals and humans. This approach reveals significant entanglements at the sensory and cognitive levels, avoiding reliance on oversimplified metaphors about molecular or chemical processes when precise tracking of such mechanisms is unfeasible. By focusing on embodied relationships, our work highlights the complex interplay of physicality and meaning in human–other taxa interactions.

## Introduction

Who are leeches? Slimy, loathful creatures that suck our blood or important actants in the environments they live in? Commodified animals one may earn money with or rather pets you can enter into an affective relationship with? These questions may be answered by adding yet another question: who are leeches in relation with whom?

The documented relationships between humans and leeches in the field of healing span over thousands of years [1]. While most people rarely interact directly with leeches, several tons are harvested annually for use in the pharmaceutical industry, with demand continuing to grow steadily [2]. The relationships between leeches and humans are, therefore, a compelling subject for exploration within the framework of more-than-human anthropology.

Our research is based on researches’ bodily experiences with leeches 2019–2024 and ethnographic fieldwork among Dagestani healers and leeches they work with, living in Makhachkala, Republic of Dagestan, North Caucasus in 2019–2021 and in Yalova, Yalova Province, in Turkey in 2024, where they have migrated for economic and political reasons since 2010s.

We propose that malleability is a particularly apt term to describe human–writhing animals relations, as it captures their unique capacity for reversibility, understood after Arregui [3], and shapes the communication with humans—not only within social and cultural contexts but also in terms of their physical appearance and workings. This concept allows for a more nuanced understanding of human–leech interactions, reflecting both leeches’ social roles and bodily transformations. We ask what does the leech malleability contribute to? What does it facilitate? We show that a leech’s malleability influences the dynamics and shape of her relationships with humans as well as creates unique possibilities for humans to interact and communicate with it.

We examine the leech’s malleability on both an individual level—focusing on the relationship between an individual leech and a human—and a social or population level. We follow Anibal Arregui who suggests that living organisms do not necessarily produce environmental reversals “as species.” At times, living beings can also individually switch between different corporeal forms, affective dispositions, and behavioural habits, in order to attune to the most mundane contexts and forms of relations [3].

Our perspective bases on the established and continuously growing body of literature on the human and more-than-human relations including already classical books such as edited by Eben Kirksey “The Multispecies Salon”, “The Mushroom at the End of the World” by Anna Tsing, “When species meet” by Donna Haraway. We adopt a relational perspective, acknowledging complexity that points to the overlapping and inter-connected character of human and non-human systems [4]. We treat leeches as actants on equal terms, but we describe the relationship from the perspective available to us through our bodily experience, observations, and conversations with healers. Close bodily contact is particularly crucial on the individual-to-individual level, where the leech’s malleability becomes more apparent and easier to perceive. We limit our focus to the human blood-sucking leeches in the context of healing and beauty business practices in Dagestan and among Dagestanis in Turkey.

## Background

The histories of leeches and humans have co-created and influenced each other for centuries [1]. This historical entanglement is evident in nearly every article discussing medicinal leeches. They often begin by referencing the presence of records on hirudotherapy in ancient cultural texts from the Mediterranean region [5–7], for example Egyptian depictions of leeches, or the writings of Ibn-Sina (Avicenna) or Hippocrates. While their work shaped European medical knowledge and might have also influenced our interlocutors cf. [8], the traditional use of leeches in Dagestan, given its location, may also reflect direct influence from Persian and Arabic sources, with records dating back to the first century [9] or even early stage of the Old Babylonian period (roughly 2000–1600 BCE) [10]. In later centuries, as Dagestan became part of the Russian Empire and then the Soviet Union, Dagestani leech therapy was influenced by broader trends in the Empire, where hirudotherapy had been prominent since at least the nineteenth century [9, 11].

In the early nineteenth century, annual leech consumption in Russia reached about 30 million, while in France, it was even higher at 100 million [9]. Sardinian merchant Pietro Battso exploited leech-rich areas in Yerevan Uezd, shipping approximately 680 kg of leeches to France in 1844. The Caucasus emerged as a significant source of leeches for Europe. In the nineteenth century, rising demand made leeches vital to rural economies in the region and other areas with unexploited wild medicinal leech populations, notably in the Ottoman Empire [12, 13]. This demand led to the emergence of professional leech gatherers [12] and marked a pivotal moment in the commodification of leeches. Sergiei Saluschev even called them the “unsung heroes of global capitalism” [12]. Overexploitation greatly depleted local leech populations in nineteenth-century Europe and the Ottoman Empire [11, 13]. This prompted legal regulations on leech harvesting [13] and the publication of practical leech farming manuals in languages such as French, German, and Swedish [11].

Leech therapy was an integral part of official medicine in the Soviet Union [14, 15], with leeches even used in a 1953 attempt to treat the dying Joseph Stalin by placing them in his ears [1, 15]. Leeches continued to play a significant role in the region's medical landscape throughout the Soviet regime and after its collapse.

In the contemporary North Caucasus, as in the entire Russian Federation, medicinal leeches (primarily *Hirudo medicinalis*) are available in pharmacies, supplied by specialized leech farms [14, 16] and broadly applied in medical, beauty-centres or Islamic healing facilities. In the 2010s, Islamic healing practices gained popularity in Dagestan, alongside a growing interest in Islam in the North Caucasus. While hijama (wet cupping), jinn exorcisms, and the use of honey and black cumin (*Nigella sativa*) were commonly discussed treatments [17], local Islamic wellness centres, clinics, and individual healers also offered leech therapy. Leech therapy has also been applied during COVID-19 pandemic as an additional measure to ease the symptoms and aftereffects. Unlike distinctly Islamic treatments, leech therapy is popular—or at least familiar—among both religious and non-religious members of the population, many of whom remember their parents or grandparents using leeches either at home or in clinics during the Soviet and post-Soviet eras, often passing the knowledge from generation to generation. Such transfer is much less likely in Turkey, due to Ataturk’s modernization reforms that have viewed leeches as un-modern cf. [18]. While in migration or exile in Turkey Dagestanis often capitalize this knowledge working with leeches or doing hijama often without official permits or licenses.

Overall, the historical presence of leeches in the everyday practices and economy continues to shape the contemporary entanglements of healers, leeches and current healing trends both in Dagestan and among Dagestanis in Turkey. In many cultural contexts, such a long and sustained leech–human interactions resulted in the presence of leech in the similes and metaphors common both in everyday language and poetry [1, 19–21]. The ambiguity of leech is present in many of them being both healing and frightening and parasitic. For example in English the word “leech” came into use early in the history of the language and had two distinct meanings: the medical practitioner and the blood-sucking worm, but their etymology was different. The Old English “laece”, meaning the worm, was related in its origin with Middle Dutch lieke, or leech. The other Old English word “laece,” meaning physician, came from Germanic languages, including Old Frisian *letza*, meaning physician, Old Saxon *laki*, and Old High German *lakki* [22]. Blood-sucking behaviour of leeches was a reason for associating them with capitalist exploitation, especially in French context, but not only [21]. Similarly in Russian—*lingua franca* of our research—word leech (Rus. *pijavka*, *пиявкa*) may mean a person that is greedy, cruel is but also negligible, the one who is living at the expense of others and therefore causing disapproving emotions [23]. Although such associations are common in many languages, leeches may also be source of positive similes and metaphors, as it is in Tatar language where leech is associated with healthy or well-fed person [24].

### Malleability of Leeches in anatomy and environment

The leech’s anatomy and morphology are crucial to its body’s malleability. We consciously point to the leeches otherness, but in consequence, similarly to Hovorka, we see the relational emergence of animal’s identity [25]. All leech species possess a hydrostatic skeleton, which allows them to move in a wiggly manner by changing the pressure of the fluid within specific segments of their segmented bodies. This mechanism also enables significant changes in the leech’s body size [26]. Leeches can enlarge their bodies by approximately ten times [26]. The digestive tract of blood-feeding leeches has special extensions to maximize the blood absorption that is why *H. medicinalis* may intake 900 per cent of their body weight during one feeding [27].

There are about 700 scientifically described species of leeches worldwide [27], though it is likely that many more species have yet to be described. Of these 700 species, 15 are used medically on a global scale [28]. Medicinal leeches represent taxa that feed on human blood. However, not all leech species consume blood; many are predatory. Among the blood-feeding species, not all target humans—some prefer hosts such as water birds (fowl), fish, turtles, or other vertebrates over mammals. Additionally, such species as *Limnatis nilotica* found in Dagestan and Turkey feeding on mammalian blood extracts it through mucous membranes rather than through the skin [28, 29].

According to the literature, three species of leeches are used medicinally in Turkey [28]: *Hirudo sulukii* (endemic to Southeastern Anatolia, with its biology still poorly understood), *Hirudo verbana* (widely distributed across Eurasia), and *Hirudo medicinalis* (found throughout Eurasia and introduced to North America) [30]. Only *H. medicinalis* is mentioned in sources on medicinal leech therapy in Dagestan. However, it is highly likely that *Hirudo orientalis* (recognized as a separate species in the early 2000s and easily confused with *H. medicinalis* [31]) and *Hirudo verbana* also function as both commodities and therapeutic partners in the region cf. [11].

Globally, since the 2000s, the demand for medicinal leeches has been increasing [14], prompting a rise in research exploring new sources of medicinal leeches. Scientists are evaluating the feasibility of culturing previously uncultured leech species and conducting in-depth studies of leech reproductive cycles [30, 32–34]. Leeches are, however, far more than just animals valued by humans for their medical properties; they are important actants in the freshwater ecosystems they inhabit. They comprise a significant portion of the diets of fish living in those habitats [35] and serve as prey for other leech species, birds, crayfish, and aquatic insects [26]. The loss of leech populations, therefore, poses a threat to the ecosystems they help to sustain. It has to be mentioned that *H. medicinalis* is already listed as Near Threatened on the IUCN Red List (IUCN Red List, 2018) and also since 1987 protected under the CITES (the Convention on International Trade in Endangered Species of Wild Fauna and Flora), because of the commercial exploitation of medicinal leech species, both historically and in modern times, together with habitat loss and climate change influencing the condition of wetlands [2, 16].

In our analysis below we have chosen not to use the term "species" and instead apply the term "taxa" as we want to emphasize the challenges associated with species as a concept cf. [36, 37]. Following Anibal Arregui's terminology of inter- and infra-species, we have chosen to adapt these terms to inter- and infra-taxa [3]. The determination of a species may be important for conservation purposes, although biologists also debate the relevance of this category [38]. Moreover, as Sket and Trontelj pointed out: it “become clear that most commercially used leeches are not the species officially declared (*H. medicinalis*), but rather its congener *H. verbana* or sometimes *H. orientalis*.” [39].

In ecological studies, it is increasingly common to encounter functional aggregations of taxa [40]. In our study, we also chose to consider leeches as an aggregation of leech taxa involved in leech therapy in both Dagestan and Turkey, collectively referring to them as medicinal leeches. We regard them as individuals participating in the research, while focusing on their bodily performance rather than their classification within a specific taxonomical category. This approach aligns with the perspective of our human interlocutors.

## Methods

Following both leeches and our Dagestani interlocutors from the North Caucasus to Turkey, we resorted to “immersive duo-ethnography”. Duo-ethnography is a collaborative research methodology that encourages joint fieldwork, as well as discussion and analysis to untangle meanings behind particular issues [41]. We use the term "immersive duo-ethnography" to highlight the deeply immersive nature of our collaborative fieldwork. While immersion is common in ethnography, it was central to our approach. To explore human–leech relationships, we relied on active, self-aware, embodied, and multisensory participation, in line with Sarah Pink’s emphasis on sensory ethnography as a way of knowing and engaging with the field [42]. We focused on touch, sounds, smells, and movement, using our bodies as experimental settings, often challenging our boundaries of disgust, pain, or safety. Our "immersive duo-ethnography" involved healing procedures on ourselves, such as applying leeches (Iwa), learning under healer supervision (Iwona), and undergoing blood cupping (Iwa), jinn exorcisms or fortunetelling. The "duo" aspect enriched our bodily experiences and provided an external perspective on/from our partner involved in classic participant observation. This approach aligns with Hovorka's call to emphasize bodily experiences in human–animal interaction research and to use diverse methods and tools to better capture animal experiences [25]. The method presents several limitations. It is time-consuming, which generally confines its application to a limited number of cases, thereby heightening the risk of bias. Effective implementation also requires prior fieldwork experience; for this reason, we do not recommend it for novice researchers. On the one hand, researchers must establish trust to gain access to participation, observation, and knowledge-sharing with healers. Becoming an apprentice takes time and multiple visits in the field. On the other hand, they must remain acutely aware of power dynamics, prioritize the well-being and potential vulnerability of participants, and engage in critical reflexivity regarding how their presence and actions influence the research process and its outcomes.

As an aside, it may be mentioned that in Nietzsche's famous text "Thus Spoke Zarathustra" the description of the encounter of Zarathustra with the “spiritually conscientious one,” refers to an attempt to understand the mind of a leech through the observation and bodily experience of blood suckling animal. According to Deleuze’s account of apprenticeship, we learn to know the world just like Nietzsche’s conscientious learns to know the world of the leech: by submitting ourselves to it, plugging into it [43].

This method intensifies the tension between participation and observation, as it often involves interventions on one’s own body and may provoke unexpected bodily reactions. For this very reason, we advocate a "duo" approach to participation, wherein one researcher undergoes the embodied intervention while the other adopts a more conventional participant–observation role. In some cases, the method also entails health risks; if procedures are conducted in unsanitary conditions, researchers may be exposed to illness or infection.

We recorded videos of healer–leech interactions, conducted ethnographic interviews exploring the knowledge of human animal. As women, we had closer access to female healers (including the two who are key interlocutors in this research), but also interviewed 8 male healers among our 26 interlocutors in Dagestan, deepening our understanding of local healing practices. The field material used in this paper comes from fieldwork conducted in 2019 and 2021 in Dagestan and in 2024 in Turkey. With the three female healers, we developed kind of apprenticeship relation, which involves multiple visits and long-term engagement (cf. [44]). However, since 2004 (Iwona) and 2008 (Iwa) conducted fieldwork in Dagestan, with the focus on Islamic healing practices and fortunetellers since 2010 which provided us with background knowledge on the subject. Initially, we focused on healers in Dagestan. However, Russia’s 2022 invasion of Ukraine forced us to shift focus to Dagestani healers who migrated to Turkey (Yalova Province – an important province for Dagestani migrants) but maintain ties with Dagestan by sending medicines, plants, talking to relatives, or conducting business.

All of the interlocutors gave their consent to participate and publish the data and photographs.

## Results

The following two excerpts from fieldnotes and audio or video recordings have been only lightly edited, with the aim of conveying the embodied experience of the relationship between leeches and researchers as well as providing details about how our duo-ethnography was conducted so that it can help other researchers in applying such a methodology in similar empirical contexts. We focus here on two key interlocutors.

**The “beauties”.** We enter a spacious room lined with shelves filled with bottles of creams and liquids, each labelled with self-printed names. Mariam, the owner of the *Center for Prophet’s Medicine: Mariam. Handmade*, greets us. In her early 50 s, she wears a pink hijab, while her assistant, Patimat, in her 40 s, dons a black-and-white hijab. Mariam shares her story, focusing on her recovery from a life-threatening condition through leech therapy and jinn exorcisms.

“Do you mind if I lay down for five minutes? asks Gurizada, our friend and door-keeper in her 40 s. “I can’t sit for long; my spine is fractured,”. Watching her stand slowly Patimat observes “She needs leech therapy.” Iwa, suffering from migraines, is also offered the treatment. Both women lay down, heads covered with yellow medical caps for the treatment. Patimat asks Iwona if she wants to apply a leech on Gurizada’s back. Despite her disgust Iwona turns on a fieldwork machismo-mode and agrees.

“Should I keep the injector until the leech attaches?” Iwona is relieved that the injector allows her to avoid direct contact with the leech. “The injector ensures they don’t wander back and forth,” Patimat explains, but she later admits, “They won’t sit just anywhere.” Watching one leech crawl on Gurizada’s back, she adds, “We need to let her find her spot on the spine – she’s searching for where the dirty blood is.” With patience, she continues, “They’re slow at first, but then they start working.” After a short while Gurizada wonders: “Maybe she does not want to eat… they need to be forced to start eating—are you feeding them something?” Patimat shakes her head.“Aaa! They’re biting me!”, shouts Gurizada to everyone’s amusement.

**Fig. 1:**
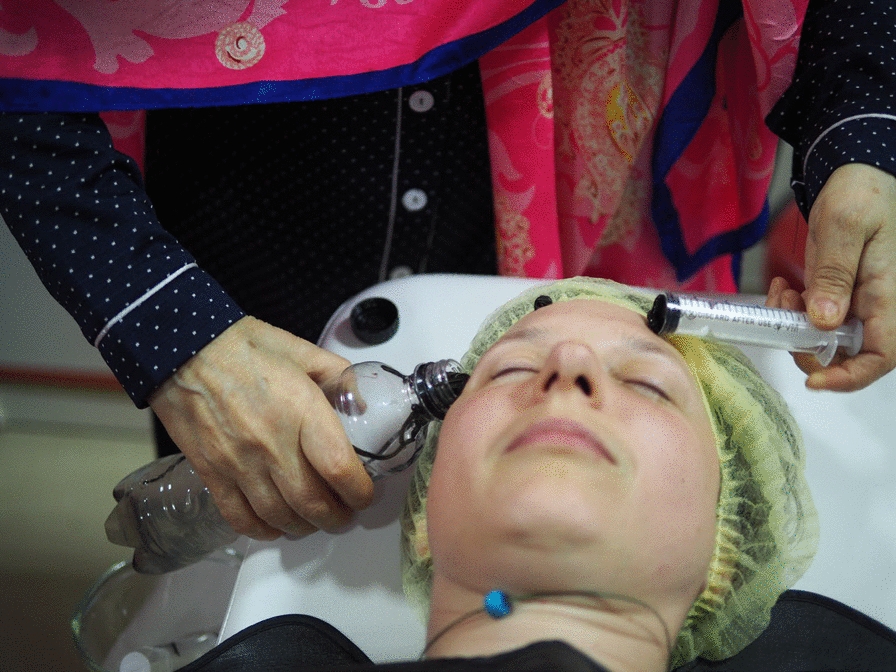
Mariam placing the leeches

Meanwhile, Mariam attempts to apply leeches to Iwa’s forehead (Fig. 1.). She places the bottle near Iwa’s head on one side while using the injector on the other. Eventually, five leeches seem to attach. When Mariam disappears into the backroom Iwa feels one cold, slimy body crawling on her forehead, probably searching for a new spot. The leech slides down beside Iwa’s ear. “She’s going to crawl into your ear now,” Iwona quips, imagining the slimy creature sliding into the ear canal. Hearing the commotion, Mariam shouts instructions from the backroom. “Use the injector to put her back!” Iwona tries but struggles as the leech wobbles and wiggles, repeatedly rolling down Iwa’s temple. To Iwona’s relief, Mariam returns and takes over: “come here… If you don’t want to stick here, no problem… Well, here you go,” Mariam says gently. “These haven’t settled yet; they’re still walking,” she notes with patience. “Soon, we’ll put the beauties where they belong. Where can they go, after all? They don’t know how persistent I am.”

“They’re animals; there’s a contact with them,” remarks Iwa. “Yes, they know I love them very much,” Mariam responds with a smile. “They’re actually quite pleasant when they crawl,” Iwa admits, softening towards the leeches.

“That one drank a lot! Look, she’s releasing liquid,” Mariam comments as Iwona marvels at the now-bloated size of one leech.

“Can you let them go in the lake after the procedure?” asks Iwa. “No, nowadays there is so much of everything, people carry so many illnesses. But you should not kill them…. I flush them down the toilet”—says Mariam visibly saddened and uneasy by this fact.

**Three leeches for 360 liras.** We enter a shop filled with the aromas of dried herbs, spices, and incense. Amina, a Dagestani woman in her late 40 s, guides us to a stall nestled among shelves stocked with herbal teas, exfoliating soaps, and natural medicines. She has been living in Yalova for the past decade. Her grandmother, back in Dagestan, was a local healer (*Rus. znakharka*), and Amina grew up observing her practices, which often included the use of leeches. Initially repelled, Amina overcame her aversion and began performing the procedures herself. She remembers when back in Dagestan she kept previously used leeches in a jar. “It didn’t look good on the windowsill”, she laughs, “Guests kept coming over and it was such an odd sight.” Today, in Yalova Amina unofficially provides leech therapy and *hijama* (blood cupping) to Muslims from the post-Soviet space.

**Fig. 2:**
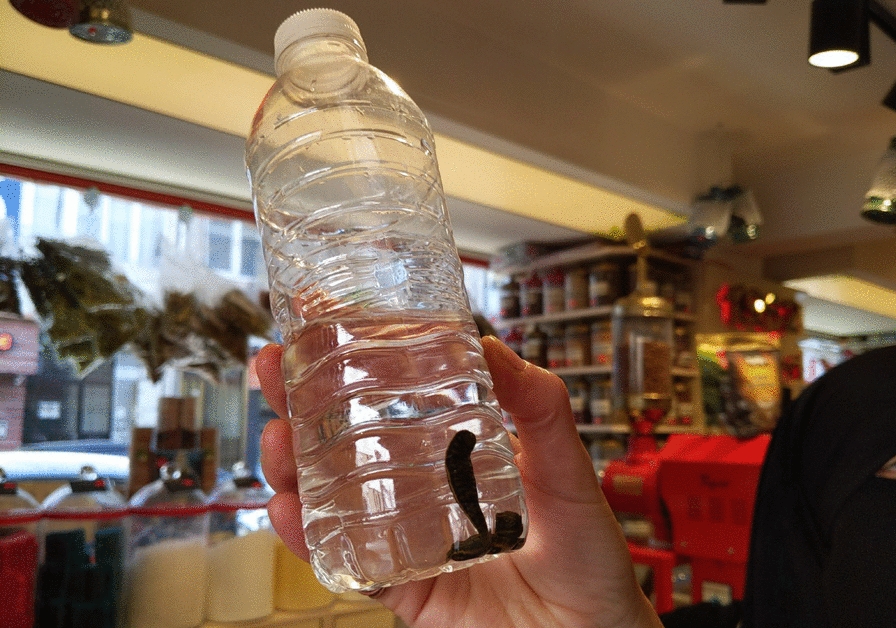
Contemplating leeches in the shop

In the shop, leeches are stored in small jars or bottles (Fig. 2), neatly categorized by size, displayed on the shelves. A nearby leaflet informs customers that leeches are not sold individually; they must purchase an entire jar containing a set number of leeches. “Look, this is 360 Turkish liras! Three leeches for 360 liras,” Amina exclaims and adds that 50 to 60 leeches are needed for a session. “Such disgusting stuff, and it costs 360 liras…” Iwona remarks, her tone a mix of amazement and irony.

“Look at how beautiful they are,” Iwa says, leaning closer to examine the jars. “Indeed, they are very beautiful. Have you noticed the intricate patterns on their bodies?” Amina points to one of the jars. The two women spend several moments closely observing the leeches, exchanging delighted comments.

Due to the high cost of leeches, Amina avoids purchasing them from stores and instead orders them online from Roma people. Amina ensures her clients prepay for their treatments. After a session, the leeches should not be re-used on different people. “When I was a child, I saw my grandmother kill leeches with salt. They suffered for two days before they died. I don’t want to torment them—it’s a sin,” Amina explains. When she first moved to Turkey, she released leeches into the canal, but over time, she began caring for them. Now, she only releases those that have grown too large to be used in therapy.

Through years of experience, Amina has learned that the purification process for leeches takes much longer than the 3–4 months often claimed in YouTube videos. “It depends on the size of the leech and how much blood it has consumed,” she says. Medium and larger leeches, in her experience, may take up to a year to cleanse themselves, while smaller ones become hungry sooner. “Only hungry leeches will bite. Those that have recently fed won’t, which makes the procedure safe.”

Still, she acknowledges the potential risks. “Leeches caught in wetlands might feed on animals like cows or dogs that wander into the bog, and they could transmit diseases like brucellosis,” Amina warns. “They can become vectors. But over time, they purify themselves—they vomit, they self-cleanse—and then they become safe to use.” Amina reuses leeches for both ethical and economic reasons.

## Analysis and discussion

### Malleability as a reversibility facilitator

What does the malleability of the leech contribute to? As will become evident from our analysis, the malleability of the leech contributes to—or rather facilitates—its reversibility (the term used after [3].

Leeches’ roles are fluidly shifted: the same human individual can perceive leeches as near-companions or coworkers in one context and as commodities or tools for work in another. They may be seen as simultaneously beautiful or ugly and disgusting, as co-healers or as objects to dispose of. Their flexible, agile bodies, combined with the roles they fulfil, contribute to this multifaceted perception and relation. While various factors contribute to leeches’ reversibility, we identify malleability as its important facilitator.

Leeches malleability facilitates their performance—their agile bodies enabled them to crawl to find a “smart” spot containing dirty blood. The malleable body of a leech that was growing when sucking patient’s blood was an indicator of the suitability of that particular leech— individual for a given treatment (similar observation in [16]). Small ones—that may eat less and make a smaller cut (with little risk of leaving a scar), were applied, for example, on a face.

The long history of leech use in medicine, as described in the background, highlights a form of inter-taxa collaboration, albeit one primarily aligned with human needs. As observed in the case studies above, leeches were often viewed as commodities by Dagestani healers we talked to. They were sold in wellness shops, such as those we visited in Yalova, displayed in open-sight in jars with water but no place to hide, with price tags based on their quantity and size, alongside other commodities like soaps or herbs. Storage and display of leeches was not any source of distress to our interlocutors, the only distress came from the high prices of leaches. As Amina mentioned, they were also sold and marketed on Instagram using stock images that disregarded their sizes and taxonomic affiliation. In these market contexts, healers’ narratives often shifted to commodity-related aspects, such as price, quantity, and size.

Leeches also serve as a means to perform specific tasks. As Amina and Mariam explained, “They need to be cleansed in order to work." These animals were thus treated to some extent as working animals—diligent but replaceable, valued primarily for their performance. They were kept in jars with previously boiled water that was periodically changed, with little regard for their natural habitats—unlike invertebrate-pets kept in terraria, where owners attempt to replicate the animals' natural environment, although they also, for various reasons, consider it necessary to keep the environment of their invertebrates disinfected [45]. The primary goal of keeping leeches in this context was to ensure they are purified for future use, thereby avoiding the need to make a decision to kill them. Leeches’ working environment alienated them from the environment they come from. In contrast in the research of Lisa Allette Brooks in the Ayurvedic leech therapy clinic in India, leeches, until they had their patients, were kept in a former turtle tank where there were plants and places to hide [16].

From being perceived as workers/working animals or commodities, leeches could shift to being seen almost as pet-like creatures, admired and respected. Mariam, for instance, shifted easily from encouraging leeches to work to admiring their agile bodies and speaking of them with love and compassion. Amina described them as smart animals with beautiful, patterned bodies. Although it may be debated whether emphasizing the visual or aesthetic value of an animal is a fully equitable and respectful way of engaging with living beings, one must acknowledge that it is not so different from how other people are perceived. This perspective may also extend to how the observing subject views themselves. These does not make it more body-positive but makes it more horizontal simultaneously pointing to various power relations particularly embedded in gender and sexuality cf. [46].

Anthropologists have studies working animals in more-than-human perspective highlighting, for example, the importance of classifying working animals and considering animal welfare and agency in various roles such as "assistance animal", "companion animal", and "justice facility animal", in the typology of the working animals they have created, they, however, did not take into account invertebrates, when creating classification focused on the purpose of the animals and the level of training they require [47]. Status of invertebrates as working animals (co-healers, companions, collection animals etc.) is complicated. On the one hand, their work is acknowledged, for example, in medical therapy to heal wounds (e.g. greenbottle blowfly (*Lucilia sericata*) maggots), in water-cleaning stations (e.g. swollen river mussels (*Unio tumidus*)), in compost (e.g. earthworms), food production (e.g. bees) or dengue and malaria-eradication (genetically modified male mosquitos). On the other, they are not included in international ethics codexes on animal welfare [48], and for example when in 2004 U.S. Food and Drug Administration included leech therapy as an official recommended treatment, it gave *H. medicinalis* a status of medical device [49]. Although the status of leech is flexible/malleable for our interlocutors, they are never just a “device”.

From the human perspective, their malleability contributed to the attractiveness of these animals as co-workers/collaborators in therapy process, whose nimble bodies could change shape, grow, and crawl. At the same time, the malleability provoked disgust. Healers admitted to being repelled by leeches in the past. Amina, for example, never thought that she herself could deal with leeches—with time the feeling of disgust faded, but when keeping leeches in the jar on her windowsill in the full sight she was quick to notice that they made an odd impression on the guests at her home. Moreover, it is reported that disgust may significantly decrease due to the prolonged physical contact with animals previously seen as disgusting [50]. In such a way, Amina changed her attitude towards the leeches and now is not any more repelled by their look she works with them and keeps them at home. To the untrained human observer, the wobbly and elongated body of a leech appears to lack clear distinctions such as up and down, or front and back. Iwona, when applying a leech on Gurizada’s back asked Patimat where is the leeches’ mouth, as on the first sight it could be both sides. Its absence of legs further enhances its capacity for reversal, both on physical and semantic levels.

Our interlocutors expressed unease with the killing of leeches, perceiving it as a sinful act within an Islamic moral framework. Although their views were not grounded in formal religious scholarship or detailed knowledge of Qur’anic exegesis or Islamic jurisprudence, they reflected a broader, tradition-based ethical sensibility—namely, that animals should not be killed except for legitimate halal purposes, such as food consumption. This ethical orientation is consistent with Islamic teachings that, among other principles, prohibit unjustified cruelty to animals. Such prohibitions are evident in Hadith literature, including the condemnation of neglect (Sahih al-Bukhari, Hadith 2365) and the proscription against killing animals for sport (Sahih Muslim, Hadith 1958). Of course, Islamic animal ethics is considerably more complex, as explored in depth by Sarra Tlili, who situates Islamic perspectives within broader debates on animal ethics and critically examines the Western biases that often shape their reception [51].

However, while our interlocutors expressed the distress with the killing, at the same time, the leeches they did not keep or let out to the canal were flushed down the toilet—the malleability of the leech, her slimy body seemingly fitting the environment of toilet water which was, possibly, resembling the imagined habitat of the leeches, enabled the cognitive dissonance of not actively killing them (for example, with salt as did Amina’s grandmother), but at the same time getting rid of them. Such ethical problems were not highlighted in Brooks’ research, leaving it unclear whether they were absent or simply excluded from the author’s analysis [16]. Brooks quotes a physician describing the process of sorting leeches purchased from a collector, which includes crushing and burying "venomous leeches" [16]. Our interlocutors undoubtedly treated leeches as sentient beings, but the decision not to kill them made on the religious grounds does not necessarily implicate the empathy towards the animal, but might have rather been just a moral stand towards the sentient being. Methodologically we opted for acceptance of our interlocutors’ ethical considerations towards leeches.

When letting out leaches to the water reservoirs healers did not consider particular leach niche preferences. Although our human interlocutors had close bodily contact with leeches they worked with, they did not engage with leeches in their natural environments, but interacted solely with individual leeches purchased from pharmacies, wellness shops, or online. That is why healers do not tend to think about leaches as diverse animal taxa inhabiting various environments, they rather looked at their malleable bodies primarily through the prism of their medicinal use. They noted differences in the size of the leech, as this factor influenced the therapeutic procedure. They did not demonstrate detailed morphological knowledge, nor did they focus on such characteristics like colour. To consider the necessity of precise taxonomical determination, it is essential to have the knowledge that there is a lot of leech taxa with diverse “life styles” and adaptations, inhabiting various ecological niches cf. [52]. Lack of such experience resulted in not noticing the malleability of leach as taxa inhabiting various environments. Moreover, this lack of experience contributed to the fact that our interlocutors did not see the leeches as pests.

Animals that induce disgust are often culturally associated with dirtiness or/and danger [53, 54]. The disgust-inducing malleability of the leech may be indirectly connected—perhaps even at an evolutionary level [55, 56]—with the perception of risks they potentially carry, risk of which healers were aware. Obviously, various other factors except the malleability of leech might influence the feelings of disgust and fear evoked by them in humans, e.g., fear of being bitten, fear of bleeding, fear of animals that may be vectors transferring various pathogenic microorganisms cf. [50]. Due to these factors, they may be seen as pests, but we did not observe malleability contributing to the classification of leeches as pests.

At the same time, cultural perceptions are never uniform or stable. Over the centuries, changes in the human attitude towards leeches’ blood-sucking behaviour significantly affected attitudes to these writhing animals. In nineteenth century when leech therapy and blood-letting was much valued healing practice in Europe leeches inspired fashion, females wore imitation leech decorations; ‘fastidious ladies used to deck their dresses with embroidered leeches’ [57]. That is why, for example, Kirk and Pembelton argue that the disgust evoked by leech cannot be seen as primordial [1]. Among religious part of the population of Dagestan blood-letting (hidjama) is a widely accepted practice to “get rid of the dirty blood”, dealing with blood is therefore familiarized—it is often talked about in casual conversations in public places (not only our interlocutors but also random people we met for example on train would discuss bloody traces they have from hidjama or dwell upon blood-letting or leeching benefits). In such a familiarized context, leech’s body growing/bloating with dirty blood is not necessarily seen as disgusting.

Summing up, we would say that malleability of the leech captures its capacity for reversibility in regard to social and cultural contexts as well as in terms of their physical appearance and the work they do. Unlike scholars focusing on inter-species entanglements—like for example Lisa Allette Brooks, who is underlining the inter-relativity within the clinic she conducts her research in on Ayurvedic leech therapy—we focused on the entanglements related to the individual body with its specifics [16]. Such an approach can possibly open up different possibility for the analysis of leech–human relations. We do not apply the pet-pest framework proposed for example by Jerolmack, as our interlocutors do not see the leeches as pests, they do not meet them in the environment, so they do not see them as a problematic animal [58].

### How leech’s malleability shapes the communication with humans

What is the role of a leech’s malleability in shaping communication? As will become evident from our analysis, the malleability of a leech enables and influences unique possibilities for humans to interact and communicate with this animal. Its malleability shapes communication in distinctive ways and contributes to the specific manner in which its communication with humans is interpreted.

In our approach, within the more-than-human framework, communication encompasses not only linguistic communication but also sensory communication, such as smells, sounds, vibrations, and visual cues that carry meaning across species [59]. It also includes behavioural signals, such as the actions and gestures of animals, as well as symbolic and spiritual connections, such as perceived communication with non-human entities like spirits, plants, or landscapes. Although in the case of communication with leeches chemical signals (chemosensation) are potentially important (cf. [60] on *bina*–plant charms), leeches insert various substances into human blood including most famously hirudin and many others (e.g. [9]), what was mentioned by our interlocutors, in this particular example, we focus on the sensory and bodily experiences of practitioners and patients. Similarly to Eva Hayward in her analysis of the interactions of population of cup corals (*Balanophyllia elegans*) from Long Marine Laboratory, marine biologists and herself, we see that the communication is possible through corporeal and sensorial capacities of organisms and their attempts to coordinate with their own specified environment [61].

The malleable body of a leech communicates with both the patient and the leech therapy practitioner through its wobbly movements, its coldness, sliminess, and the bite at the beginning of suckling, among other sensations. Those sensations have synaesthetic quality, organisms emerge for each other (e.g. leech emerges for human animal) through them [61] While there are ways a leech communicates or interacts that are not directly connected to its malleability—such as biting or expelling chemical substances—it was primarily its malleability that was most noticed and remarked upon by healers, patients, and anthropologists during their interactions with leeches. The tactile interaction was most important. Some of the leech’s actions may not have been intended as communication with the external world at all. However, our focus lies on how these actions were read and interpreted by humans. What constitutes a communicate from a leech to a human and from a human to a leech is to be interpreted in-between; the communicate is created through interaction cf. [59].

It was the leech’s malleability that enabled Patimat to poke it back and forth to check if it had fallen asleep or still wanted to suck. While doing so, she compared the leech to a tiny, wobbly infant, gently stroked on the cheek to see if they are still nursing or have fallen asleep and can now be peacefully detached. This poking caused one leech to fall off, while another “woke up” and resumed sucking. It was the leech’s malleable, cold body that shaped the way it crawled on Gurizada’s back, enabling her to feel whether the leech was “running away,” “falling off,” or, from the healer’s perspective, searching for a spot with dirty blood. Similarly it was the leech’s cold, malleable growing body on Iwa’s forehead—felt rather than seen—that allowed her to understand that the leech had completed its task. When Iwona tried to lift the leech that had fallen off Iwa’s forehead, its malleable, writhing, and flexing body seemed to convey to the inexperienced anthropologist: “Don’t touch me,” “Don’t eat me,” or “I want to run away.” These leech movements are typical for annelids and make it more difficult for a predator to catch the leech.

In a one-to-one relationship, the malleability of a leech facilitates human sensations of an interaction with a leech. When Mariam gently speaks to a leech, saying, “Come here… If you don’t want to stick here, no problem… Well, here you go,” she appears to acknowledge and accept that this particular leech might not be in the mood or the right moment for sucking. Mariam is, therefore, treating the leech as an individual being, and she is treating her with empathy and compassion—she tries to understand and share the feelings of a leech by putting herself in her position and imagining how they might feel, or react in a given situation. While empathy is a feeling often directed towards many animals, it frequently does not extend to invertebrates [62], even when they are threatened with extinction. Katie Woolaston and Afshin Akhtar-Khavari highlight this issue, calling for environmental law to prioritize empathy and compassion especially for invertebrate species facing the risk of extinction [63].

When talking to the leeches with empathy/compassion or calling leeches “my beauties” Mariam is speaking primarily to herself and her patients, creating a positive atmosphere and bringing calmness to her movements. Therefore, this cannot be framed as an inter-taxa interaction but rather as an infra-taxa interaction. This interaction might also involve chemical signals transmission from human body—though we do not know if the leech perceives these signals. But we know that, in the interaction between the healer and the patient-anthropologist, such communications convey that the wobbly, slimy body of the leech is not disgusting to the healer.

The malleability of a leech plays a key role in shaping human interaction and communication with the animal. Through its wobbly movements, coldness, sliminess, the leech conveys sensory cues to both patients and practitioners. This malleable quality influences how its actions are interpreted—whether as signals of resistance, compliance, or purpose. The leech’s malleability was particularly noted by healers, patients, and anthropologists as a primary factor in understanding its behaviour and intentions during therapy sessions. This malleability enhances the nuanced, multisensory communication between humans and leeches, demonstrating how bodily qualities can shape inter-taxa interactions.

## Conclusions

In this paper, we have introduced the term “malleability” and tried to demonstrate its usefulness in regard to human–writhing animals, on the example of leech–human relations. This term highlights relational emergence of animal identity cf. [25]. While there are other aspects of such a relationship that are certainly worth exploring, by focusing on malleability we were able to, in our analysis, highlight two key dimensions: malleability as a facilitator of reversibility and malleability as a shaper and facilitator of communication.

We have demonstrated that the malleability of the leech emerges as an important factor in understanding its multifaceted roles in shaping human-leech interactions. We have shown that leeches occupy a spectrum of roles in human perception and practice, ranging from near-companions and ethical subjects (despite not being protected by bioethical codexes for research conducting [48]) to commodities and tools for medical procedures. Their malleability—both physical and semantic – enables this fluid reversibility. The flexibility of their bodies, combined with the varied roles they fulfil, allows leeches to be admired for their beauty and work in one moment and to be disposed of as unwanted objects in another.

Religious beliefs influence the perception of animals as ethical subjects. However, the bodily malleability of leeches suggests to our interlocutors hands-on solutions to moral dilemmas.

The malleability of the leech also profoundly shapes its communication and interaction with humans. By enabling sensory and behavioural signals, the leech’s flexible body creates opportunities for nuanced, multisensory exchanges, shaping how humans interpret its actions—whether as cooperation, resistance, or purposeful behaviour. The malleability of leeches influences the sensory and emotional responses of humans, mediating both perceptions of disgust and admiration.

We clearly demonstrate that communication between an individual leech and the therapist is performed through bodily signals. Verbal signals—words spoken by the therapist—while formally directed at the leech, are in practice a communicate between the therapist and themself or patient.

It is important to unravel the verbal communicates in the relations between human and more-than-human actants, because what might initially appear to be inter-taxa communication is, often infra-taxa communication. The lack of verbal communication is obviously not meant to suggest any inferiority of a non-human actant, but rather to show how different bodies can communicate with a malleable body and that communication occurs through other means.

Overall, to answer the question, "Who is the leech?" it is essential not only to ask (as we did at the beginning) "in relation to whom," but also, "at which moment in time," "under what conditions," and "in which context." The malleability of the leech highlights how deeply contextual the answer is to what might seem like a simple question: "Who is the leech?".

It is not always possible to examine human–invertebrate relationships beyond a populational perspective. However, where feasible, we find it crucial to approach them as one-to-one bodily interactions, offering a contribution to anthropological studies on invertebrates. In research involving various actants across diverse taxa, focusing on the bodily level (sensory cognition level) of the relationship allows for revealing significant entanglements without resorting to broad metaphors based on superficial interpretations of molecular or chemical processes while there is no possibility to track and precisely interpret these processes. Research aimed at analysing these processes requires a much broader interdisciplinary approach. While such research is feasible, anthropologists often tend to superficially understand these processes and construct metaphors of relationships with more-than-human actants based on this limited understanding. Therefore, we propose focusing, especially when not working within larger transdisciplinary teams, on practices and bodily experiences that can be effectively explored using the available tools. While more research would be needed in regard to other writhing animals, we suggest that animals with writhing movements, enabled by their bodily malleability, may often be perceived as more reversible than those without such movements.

While leeches exhibit diverse species and adaptations across various ecological niches, the healers interacted only with leeches purchased from pharmacies or online, disregarding their diversification. Their focus was not on taxonomic classification or morphology but primarily on leech size, which influenced the therapeutic process.

The cultural perceptions of various animals are present in similes and metaphors [e.g. 1, 24]; therefore, language could be as well an interesting field to explore whirling animals malleability especially in multi-language environment of Dagestanis. Nevertheless, it was not in our focus as none of our interlocutors pointed to the metaphorical meaning of the leeches. Moreover, we as researchers were focused on the embodied experience of the interaction with leech not on the more discursive one. We used immersive, embodied research method involving apprenticeship.

On the methodological note, we conclude from our research process that resorting to immersive duo-ethnography that includes “using” our bodies as research tools provides opportunities to explore the non-verbal elements of interactions between writhing animals and humans. Such a methodological approach places the responsibility on researchers to determine whether to align with the ethical sensibilities of their interlocutors (as we chose to do), or, alternatively, to decline participation in practices involving animals they know will subsequently be discarded, based on their own ethical principles.

Overall, we believe that our findings may enrich the literature on more-than-human relations with malleable animals. However, we also acknowledge that our results might be influenced by the biases of the local contexts of our fieldwork and the unique characteristics of leeches as an animal. Our research highlights leeches as working animals having intrinsic value—despite the difficulty for humans to see invertebrates as such—therefore such studies or projects could contribute meaningfully to discussions about invertebrates as ethical subjects and the recognition of invertebrate rights.

## Data Availability

The data that support the findings of this study are available from the authors at Institute of Ethnology and Cultural Anthropology University of Warsaw, but restrictions apply to the availability of these data, which are sensitive data due to the personal situation of the interlocutors living in the authoritarian state, so are not publicly available. The data are, however, available from the authors upon reasonable request and with the permission of Ethical Commission of Institute of Ethnology and Cultural Anthropology University of Warsaw.
